# Case of a Tumor of the Leg, Supposed to Have Been Occasioned by a Ruptured Vein

**Published:** 1785

**Authors:** 


VI. *Case of a Tumor of the Leg, supposed to have been occasioned by a ruptured Vein. Communicated in a letter to Dr.* Simmons, F.R.S. *by Mr.* James Pascoe, *Surgeon at Tregony, in Cornwall.*

W. A. about fourteen years of age, received an injury on his left leg, by a fall, apparently so slight, that he made little or no complaint; and I apprehend it was some weeks afterwards before he shewed it to his parents, when there appeared a small, hard, and undiscoloured tumor on the tibia. Not much attention was at first paid to it, and for some weeks it was submitted to the care of the good women in the neighbourhood. A surgeon was at length consulted, who finding a tumor now grown pretty large, painful, and still indurated, tried different methods, internally and exterexternally, either to difperfe, or to bring it to fuppuration, but to no purpofe.

By this time, the boy's health began vifibly to decline, and he grew emaciated and hectic; a medical gentleman, who had vifited many feminaries of medical knowledge, not only in this kingdom, but in foreign ones, was defired to fee it; and being, I prefume, not acquainted with the caufe, and finding a very formidable tumor furrounding the whole leg, fcarcely at all inflamed, but feeling in fome parts of it foft to the touch, he expected that a very confiderable fuppuration would take place, and therefore ordered the bark in large quantities, to enable the patient to fupport the great expected difcharge. An emollient cataplafm was applied externally. About this time alfo a furgeon, of very confiderable practice in a neighbouring county, and who is a man of eminence and fkill, was confulted by letter, and recommended the application of a pretty extenfive cauftic. Bufinefs calling me to the town where the furgeon in ordinary lived, on vifiting him, he told me the cafe of this poor boy, and offered to attend me, if I had a defire to fee him. I faw him, and it was too evident at firft fight, that the only poffible chance for preferving the poor boy's boy's life, was amputation, and it ought to have been more ſtrongly enforced. I found the patient emaciated to a great degree, with a very conſiderable tumor extending all round his leg, and feeling, in ſome parts of it, as if a fluid was contained in it. The cauſtic was applied, but on its removal, no pus, only a bloody ichor was diſcharged.

The ſurgeon employed had read Mr. Balfour's caſe of a diſeaſed leg, in the fourth volume of the Medical Obſervations and Inquiries*, and thought this a ſimilar caſe; but that gentleman ſeems to me to have been quite unacquainted with the nature of the caſe he has deſcribed; which is the more to be wondered at, as a paper, by Mr. Elſe, in the third volume of the ſame work †, would have afforded him complete information. Not ſatisfied with Mr. Balfour's account, I conſulted every author in my poſſeſſion, that I thought might give me information on this ſubject. Amongſt others, I peruſed the 50th chapter of Morgagni's work *De Sedibus et Cauſis Morborum*, but could meet with nothing ſatisfactory, till I read the paper,

* Page 1.

† See Medical Obſerv. and Inq. Vol. III. p. 169.

juſt juſt now mentioned, in the third volume of the Medical Obſervations, when it appeared to me, beyond a doubt, that the poor boy's diſorder, whoſe caſe I have related, and the caſe deſcribed by Mr. Balfour, (though he ſeems to apprehend it originated from the bone) were neither more nor leſs than ruptured veins, the conſequence of the fall; and that all the miſchief was owing, in both caſes, to extravaſated blood. After this, I again viſited the patient, with his ſurgeon. The parents were averſe to amputation, and the poor boy was reduced ſo low, that he ſoon fell a victim to a diſeaſe, which, if the cauſe of it had been known in time, might have been eaſily prevented.

The ſurgeon informed me, that on diſſecting the limb, after the death of the boy, he found two inches of the fibula in a carious ſtate. The contents of the tumor were coagulated blood and ichor.

From the different caſes that came under Mr. Elſe's inſpection, it appears that the cauſe of theſe tumors was not properly known early enough to prevent caries of the bone, &c.

If you think this hiſtory may be at all conducive to engage the early attention of ſurgeons to tumors, without diſcolouration, that may gradually gradually make their appearance after blows on the leg, or other parts, be ſo kind as to procure it a place in the London Medical Journal.

QUERE. May not the ſlow progreſs of a tumor of this kind be a good criterion to determine the cauſe of it to ariſe rather from a ruptured vein than artery?

*Tregony,*

May 10th, 1785.

